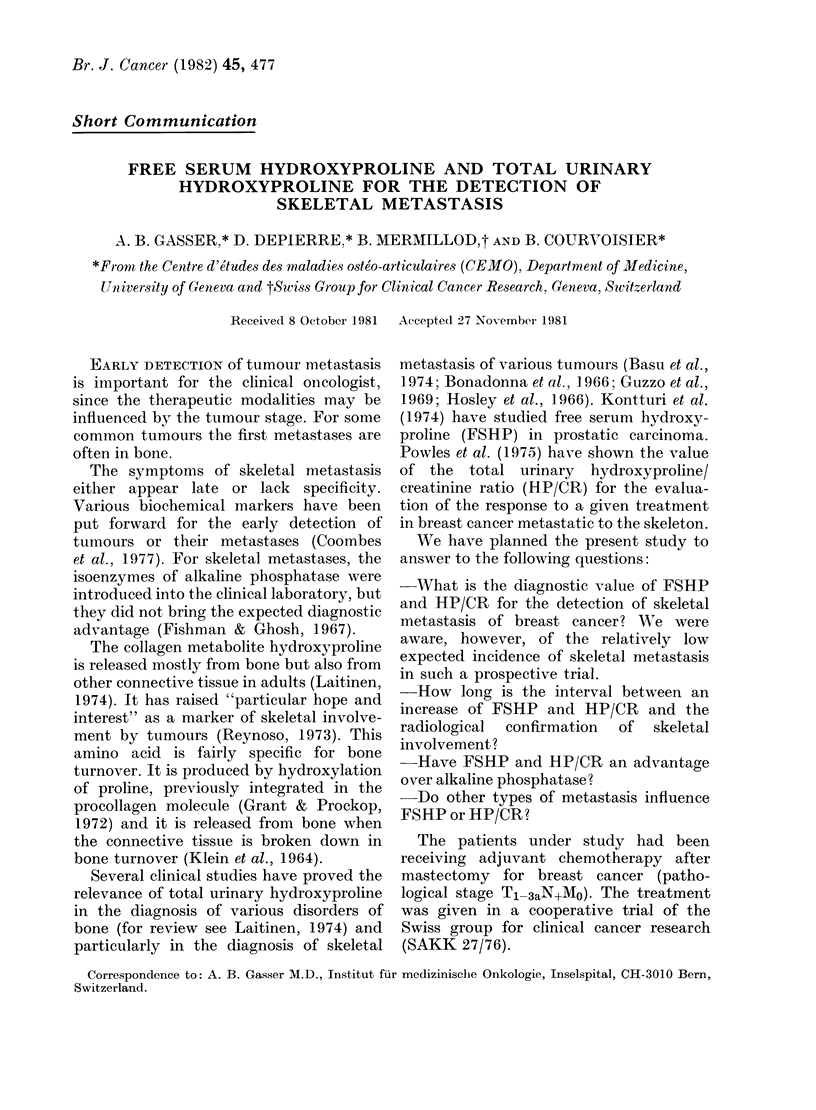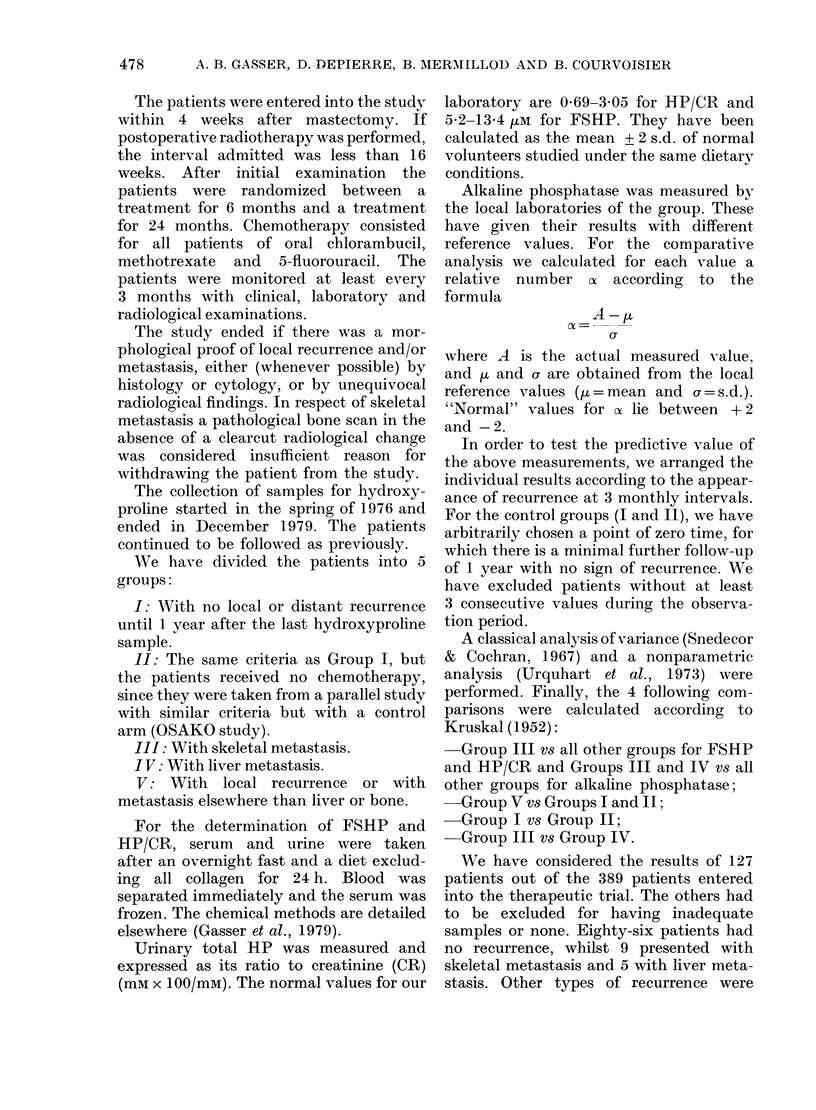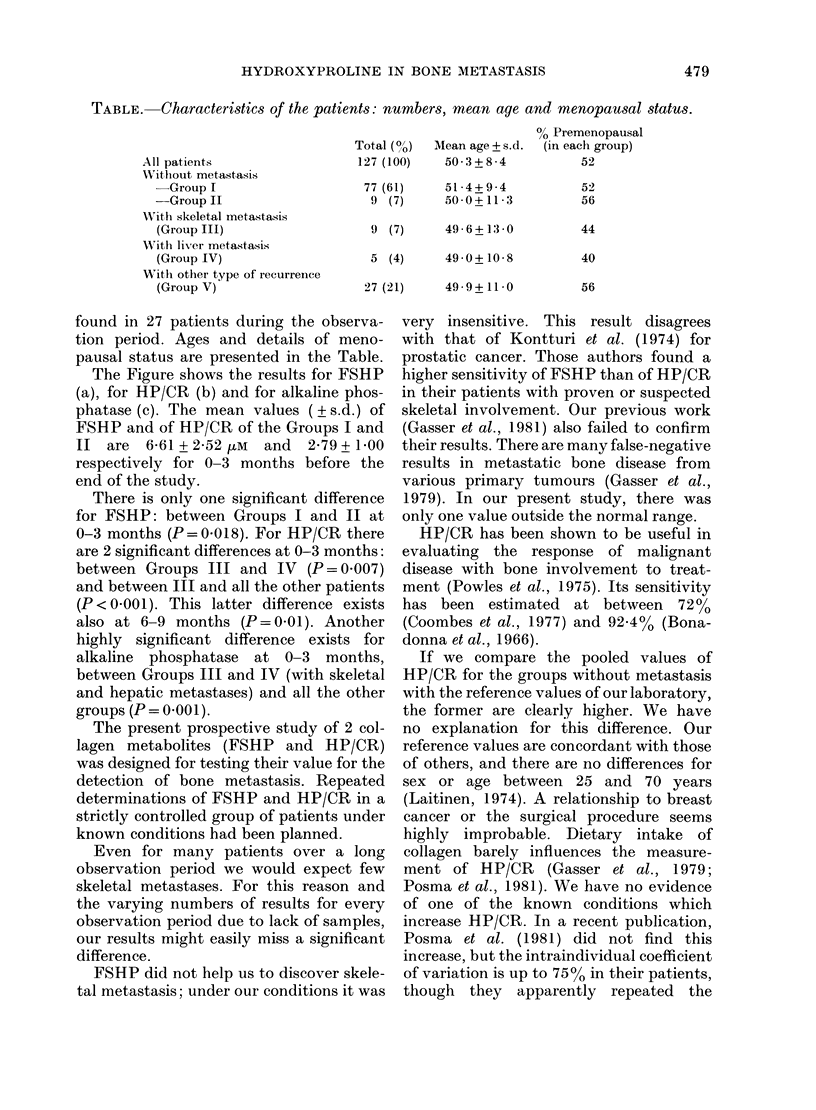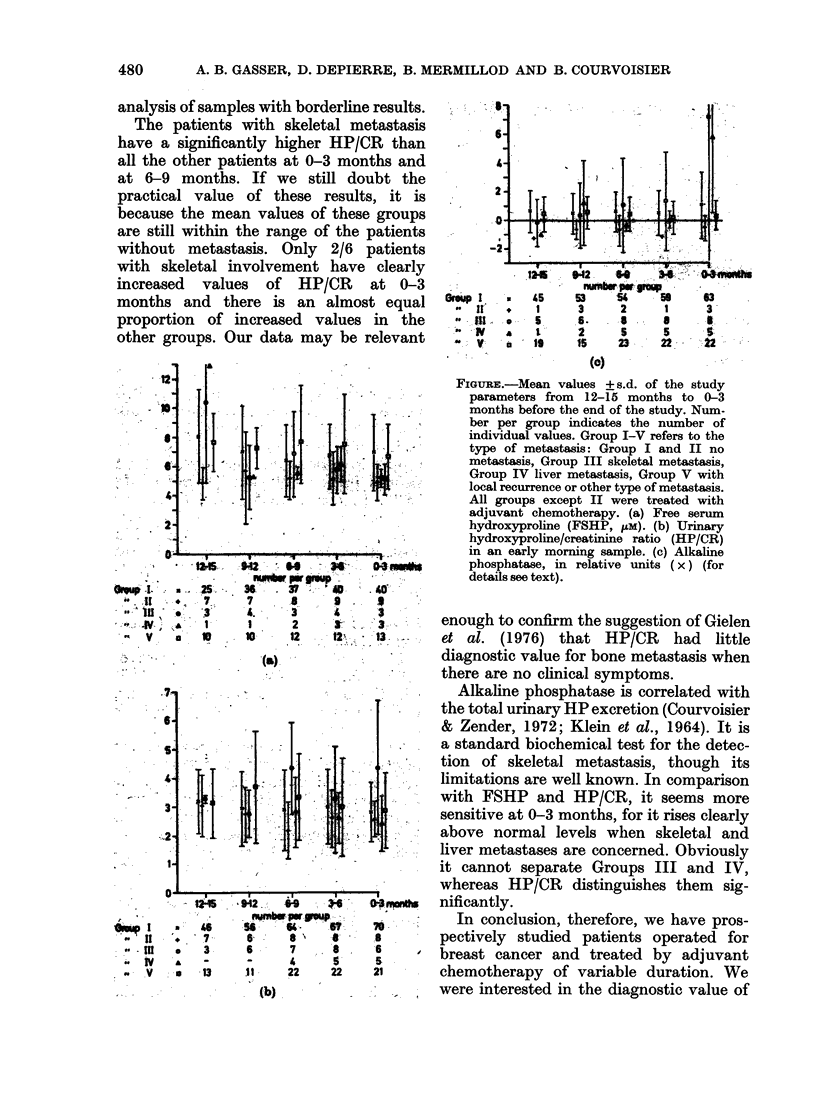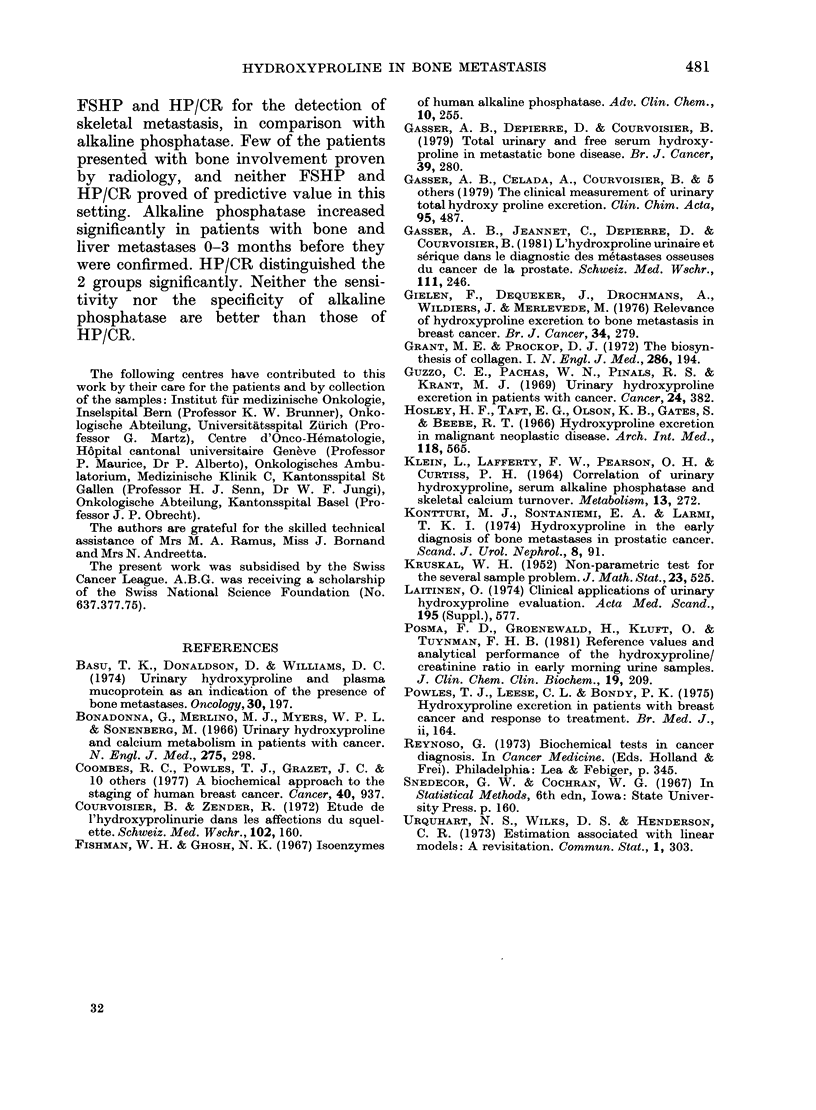# Free serum hydroxyproline and total urinary hydroxyproline for the detection of skeletal metastasis.

**DOI:** 10.1038/bjc.1982.78

**Published:** 1982-03

**Authors:** A. B. Gasser, D. Depierre, B. Mermillod, B. Courvoisier


Br. J. Cancer (1982) 45, 477

*Short Communication*

### FREE SERUM HYDROXYPROLINE AND TOTAL URINARY HYDROXYPROLINE FOR THE DETECTION OF SKELETAL METASTASIS

A. B. GASSER,* D. DEPIERRE,* B. MERMILLOD,† AND B. COURVOISIER*

**From the Centre d'études des maladies ostéo-articulaires (CEMO), Department of Medicine, University of Geneva and †Swiss Group for Clinical Cancer Research, Geneva, Switzerland*

Received 8 October 1981 Accepted 27 November 1981

EARLY DETECTION of tumour metastasis is important for the clinical oncologist, since the therapeutic modalities may be influenced by the tumour stage. For some common tumours the first metastases are often in bone.

The symptoms of skeletal metastasis either appear late or lack specificity. Various biochemical markers have been put forward for the early detection of tumours or their metastases (Coombes *et al.*, 1977). For skeletal metastases, the isoenzymes of alkaline phosphatase were introduced into the clinical laboratory, but they did not bring the expected diagnostic advantage (Fishman & Ghosh, 1967).

The collagen metabolite hydroxyproline is released mostly from bone but also from other connective tissue in adults (Laitinen, 1974). It has raised "particular hope and interest" as a marker of skeletal involvement by tumours (Reynoso, 1973). This amino acid is fairly specific for bone turnover. It is produced by hydroxylation of proline, previously integrated in the procollagen molecule (Grant & Prockop, 1972) and it is released from bone when the connective tissue is broken down in bone turnover (Klein *et al.*, 1964).

Several clinical studies have proved the relevance of total urinary hydroxyproline in the diagnosis of various disorders of bone (for review see Laitinen, 1974) and particularly in the diagnosis of skeletal metastasis of various tumours (Basu *et al.*, 1974; Bonadonna *et al.*, 1966; Guzzo *et al.*, 1969; Hosley *et al.*, 1966). Kontturi *et al.* (1974) have studied free serum hydroxyproline (FSHP) in prostatic carcinoma. Powles *et al.* (1975) have shown the value of the total urinary hydroxyproline/creatinine ratio (HP/CR) for the evaluation of the response to a given treatment in breast cancer metastatic to the skeleton.

We have planned the present study to answer to the following questions:

—What is the diagnostic value of FSHP and HP/CR for the detection of skeletal metastasis of breast cancer? We were aware, however, of the relatively low expected incidence of skeletal metastasis in such a prospective trial.
—How long is the interval between an increase of FSHP and HP/CR and the radiological confirmation of skeletal involvement?
—Have FSHP and HP/CR an advantage over alkaline phosphatase?
—Do other types of metastasis influence FSHP or HP/CR?

The patients under study had been receiving adjuvant chemotherapy after mastectomy for breast cancer (pathological stage $T_{1-3a}N_{+}M_{0}$). The treatment was given in a cooperative trial of the Swiss group for clinical cancer research (SAKK 27/76).

Correspondence to: A. B. Gasser M.D., Institut für medizinische Onkologie, Inselspital, CH-3010 Bern, Switzerland.

The patients were entered into the study within 4 weeks after mastectomy. If postoperative radiotherapy was performed, the interval admitted was less than 16 weeks. After initial examination the patients were randomized between a treatment for 6 months and a treatment for 24 months. Chemotherapy consisted for all patients of oral chlorambucil, methotrexate and 5-fluorouracil. The patients were monitored at least every 3 months with clinical, laboratory and radiological examinations.

The study ended if there was a morphological proof of local recurrence and/or metastasis, either (whenever possible) by histology or cytology, or by unequivocal radiological findings. In respect of skeletal metastasis a pathological bone scan in the absence of a clearcut radiological change was considered insufficient reason for withdrawing the patient from the study.

The collection of samples for hydroxyproline started in the spring of 1976 and ended in December 1979. The patients continued to be followed as previously.

We have divided the patients into 5 groups:

*I:* With no local or distant recurrence until 1 year after the last hydroxyproline sample.

*II:* The same criteria as Group I, but the patients received no chemotherapy, since they were taken from a parallel study with similar criteria but with a control arm (OSAKO study).

*III:* With skeletal metastasis.

*IV:* With liver metastasis.

*V:* With local recurrence or with metastasis elsewhere than liver or bone.

For the determination of FSHP and HP/CR, serum and urine were taken after an overnight fast and a diet excluding all collagen for 24 h. Blood was separated immediately and the serum was frozen. The chemical methods are detailed elsewhere (Gasser *et al.*, 1979).

Urinary total HP was measured and expressed as its ratio to creatinine (CR) (mM × 100/mM). The normal values for our laboratory are 0·69–3·05 for HP/CR and 5·2–13·4 $\mu$M for FSHP. They have been calculated as the mean $\pm 2$ s.d. of normal volunteers studied under the same dietary conditions.

Alkaline phosphatase was measured by the local laboratories of the group. These have given their results with different reference values. For the comparative analysis we calculated for each value a relative number $\alpha$ according to the formula

$$\alpha = \frac{A - \mu}{\sigma}$$

where $A$ is the actual measured value, and $\mu$ and $\sigma$ are obtained from the local reference values ($\mu$ = mean and $\sigma$ = s.d.). "Normal" values for $\alpha$ lie between $+2$ and $-2$.

In order to test the predictive value of the above measurements, we arranged the individual results according to the appearance of recurrence at 3 monthly intervals. For the control groups (I and II), we have arbitrarily chosen a point of zero time, for which there is a minimal further follow-up of 1 year with no sign of recurrence. We have excluded patients without at least 3 consecutive values during the observation period.

A classical analysis of variance (Snedecor & Cochran, 1967) and a nonparametric analysis (Urquhart *et al.*, 1973) were performed. Finally, the 4 following comparisons were calculated according to Kruskal (1952):

—Group III *vs* all other groups for FSHP and HP/CR and Groups III and IV *vs* all other groups for alkaline phosphatase;
—Group V *vs* Groups I and II;
—Group I *vs* Group II;
—Group III *vs* Group IV.

We have considered the results of 127 patients out of the 389 patients entered into the therapeutic trial. The others had to be excluded for having inadequate samples or none. Eighty-six patients had no recurrence, whilst 9 presented with skeletal metastasis and 5 with liver metastasis. Other types of recurrence were

TABLE.—*Characteristics of the patients: numbers, mean age and menopausal status.*

| | Total (%) | Mean age ± s.d. | % Premenopausal (in each group) |
|---|---|---|---|
| All patients | 127 (100) | 50·3 ± 8·4 | 52 |
| Without metastasis | | | |
| —Group I | 77 (61) | 51·4 ± 9·4 | 52 |
| —Group II | 9 (7) | 50·0 ± 11·3 | 56 |
| With skeletal metastasis (Group III) | 9 (7) | 49·6 ± 13·0 | 44 |
| With liver metastasis (Group IV) | 5 (4) | 49·0 ± 10·8 | 40 |
| With other type of recurrence (Group V) | 27 (21) | 49·9 ± 11·0 | 56 |

found in 27 patients during the observation period. Ages and details of menopausal status are presented in the Table.

The Figure shows the results for FSHP (a), for HP/CR (b) and for alkaline phosphatase (c). The mean values (± s.d.) of FSHP and of HP/CR of the Groups I and II are 6·61 ± 2·52 μM and 2·79 ± 1·00 respectively for 0–3 months before the end of the study.

There is only one significant difference for FSHP: between Groups I and II at 0–3 months ($P = 0·018$). For HP/CR there are 2 significant differences at 0–3 months: between Groups III and IV ($P = 0·007$) and between III and all the other patients ($P < 0·001$). This latter difference exists also at 6–9 months ($P = 0·01$). Another highly significant difference exists for alkaline phosphatase at 0–3 months, between Groups III and IV (with skeletal and hepatic metastases) and all the other groups ($P = 0·001$).

The present prospective study of 2 collagen metabolites (FSHP and HP/CR) was designed for testing their value for the detection of bone metastasis. Repeated determinations of FSHP and HP/CR in a strictly controlled group of patients under known conditions had been planned.

Even for many patients over a long observation period we would expect few skeletal metastases. For this reason and the varying numbers of results for every observation period due to lack of samples, our results might easily miss a significant difference.

FSHP did not help us to discover skeletal metastasis; under our conditions it was very insensitive. This result disagrees with that of Kontturi *et al.* (1974) for prostatic cancer. Those authors found a higher sensitivity of FSHP than of HP/CR in their patients with proven or suspected skeletal involvement. Our previous work (Gasser *et al.*, 1981) also failed to confirm their results. There are many false-negative results in metastatic bone disease from various primary tumours (Gasser *et al.*, 1979). In our present study, there was only one value outside the normal range.

HP/CR has been shown to be useful in evaluating the response of malignant disease with bone involvement to treatment (Powles *et al.*, 1975). Its sensitivity has been estimated at between 72% (Coombes *et al.*, 1977) and 92·4% (Bonadonna *et al.*, 1966).

If we compare the pooled values of HP/CR for the groups without metastasis with the reference values of our laboratory, the former are clearly higher. We have no explanation for this difference. Our reference values are concordant with those of others, and there are no differences for sex or age between 25 and 70 years (Laitinen, 1974). A relationship to breast cancer or the surgical procedure seems highly improbable. Dietary intake of collagen barely influences the measurement of HP/CR (Gasser *et al.*, 1979; Posma *et al.*, 1981). We have no evidence of one of the known conditions which increase HP/CR. In a recent publication, Posma *et al.* (1981) did not find this increase, but the intraindividual coefficient of variation is up to 75% in their patients, though they apparently repeated the analysis of samples with borderline results.

The patients with skeletal metastasis have a significantly higher HP/CR than all the other patients at 0–3 months and at 6–9 months. If we still doubt the practical value of these results, it is because the mean values of these groups are still within the range of the patients without metastasis. Only 2/6 patients with skeletal involvement have clearly increased values of HP/CR at 0–3 months and there is an almost equal proportion of increased values in the other groups. Our data may be relevant

(a)

(b)

(c)

FIGURE.—Mean values ±s.d. of the study parameters from 12–15 months to 0–3 months before the end of the study. Number per group indicates the number of individual values. Group I–V refers to the type of metastasis: Group I and II no metastasis, Group III skeletal metastasis, Group IV liver metastasis, Group V with local recurrence or other type of metastasis. All groups except II were treated with adjuvant chemotherapy. (a) Free serum hydroxyproline (FSHP, $\mu$M). (b) Urinary hydroxyproline/creatinine ratio (HP/CR) in an early morning sample. (c) Alkaline phosphatase, in relative units (×) (for details see text).

enough to confirm the suggestion of Gielen *et al.* (1976) that HP/CR had little diagnostic value for bone metastasis when there are no clinical symptoms.

Alkaline phosphatase is correlated with the total urinary HP excretion (Courvoisier & Zender, 1972; Klein *et al.*, 1964). It is a standard biochemical test for the detection of skeletal metastasis, though its limitations are well known. In comparison with FSHP and HP/CR, it seems more sensitive at 0–3 months, for it rises clearly above normal levels when skeletal and liver metastases are concerned. Obviously it cannot separate Groups III and IV, whereas HP/CR distinguishes them significantly.

In conclusion, therefore, we have prospectively studied patients operated for breast cancer and treated by adjuvant chemotherapy of variable duration. We were interested in the diagnostic value of

FSHP and HP/CR for the detection of skeletal metastasis, in comparison with alkaline phosphatase. Few of the patients presented with bone involvement proven by radiology, and neither FSHP and HP/CR proved of predictive value in this setting. Alkaline phosphatase increased significantly in patients with bone and liver metastases 0–3 months before they were confirmed. HP/CR distinguished the 2 groups significantly. Neither the sensitivity nor the specificity of alkaline phosphatase are better than those of HP/CR.

The following centres have contributed to this work by their care for the patients and by collection of the samples: Institut für medizinische Onkologie, Inselspital Bern (Professor K. W. Brunner), Onkologische Abteilung, Universitätsspital Zürich (Professor G. Martz), Centre d'Onco-Hématologie, Hôpital cantonal universitaire Genève (Professor P. Maurice, Dr P. Alberto), Onkologisches Ambulatorium, Medizinische Klinik C, Kantonsspital St Gallen (Professor H. J. Senn, Dr W. F. Jungi), Onkologische Abteilung, Kantonsspital Basel (Professor J. P. Obrecht).

The authors are grateful for the skilled technical assistance of Mrs M. A. Ramus, Miss J. Bornand and Mrs N. Andreetta.

The present work was subsidised by the Swiss Cancer League. A.B.G. was receiving a scholarship of the Swiss National Science Foundation (No. 637.377.75).

### REFERENCES

Basu, T. K., Donaldson, D. & Williams, D. C. (1974) Urinary hydroxyproline and plasma mucoprotein as an indication of the presence of bone metastases. *Oncology*, **30**, 197.

Bonadonna, G., Merlino, M. J., Myers, W. P. L. & Sonenberg, M. (1966) Urinary hydroxyproline and calcium metabolism in patients with cancer. *N. Engl. J. Med.*, **275**, 298.

Coombes, R. C., Powles, T. J., Grazet, J. C. & 10 others (1977) A biochemical approach to the staging of human breast cancer. *Cancer*, **40**, 937.

Courvoisier, B. & Zender, R. (1972) Etude de l'hydroxyprolinurie dans les affections du squelette. *Schweiz. Med. Wschr.*, **102**, 160.

Fishman, W. H. & Ghosh, N. K. (1967) Isoenzymes of human alkaline phosphatase. *Adv. Clin. Chem.*, **10**, 255.

Gasser, A. B., Depierre, D. & Courvoisier, B. (1979) Total urinary and free serum hydroxyproline in metastatic bone disease. *Br. J. Cancer*, **39**, 280.

Gasser, A. B., Celada, A., Courvoisier, B. & 5 others (1979) The clinical measurement of urinary total hydroxy proline excretion. *Clin. Chim. Acta*, **95**, 487.

Gasser, A. B., Jeannet, C., Depierre, D. & Courvoisier, B. (1981) L'hydroxproline urinaire et sérique dans le diagnostic des métastases osseuses du cancer de la prostate. *Schweiz. Med. Wschr.*, **111**, 246.

Gielen, F., Dequeker, J., Drochmans, A., Wildiers, J. & Merlevede, M. (1976) Relevance of hydroxyproline excretion to bone metastasis in breast cancer. *Br. J. Cancer*, **34**, 279.

Grant, M. E. & Prockop, D. J. (1972) The biosynthesis of collagen. I. *N. Engl. J. Med.*, **286**, 194.

Guzzo, C. E., Pachas, W. N., Pinals, R. S. & Krant, M. J. (1969) Urinary hydroxyproline excretion in patients with cancer. *Cancer*, **24**, 382.

Hosley, H. F., Taft, E. G., Olson, K. B., Gates, S. & Beebe, R. T. (1966) Hydroxyproline excretion in malignant neoplastic disease. *Arch. Int. Med.*, **118**, 565.

Klein, L., Lafferty, F. W., Pearson, O. H. & Curtiss, P. H. (1964) Correlation of urinary hydroxyproline, serum alkaline phosphatase and skeletal calcium turnover. *Metabolism*, **13**, 272.

Kontturi, M. J., Sontaniemi, E. A. & Larmi, T. K. I. (1974) Hydroxyproline in the early diagnosis of bone metastases in prostatic cancer. *Scand. J. Urol. Nephrol.*, **8**, 91.

Kruskal, W. H. (1952) Non-parametric test for the several sample problem. *J. Math. Stat.*, **23**, 525.

Laitinen, O. (1974) Clinical applications of urinary hydroxyproline evaluation. *Acta Med. Scand.*, **195** (Suppl.), 577.

Posma, F. D., Groenewald, H., Kluft, O. & Tuynman, F. H. B. (1981) Reference values and analytical performance of the hydroxyproline/creatinine ratio in early morning urine samples. *J. Clin. Chem. Clin. Biochem.*, **19**, 209.

Powles, T. J., Leese, C. L. & Bondy, P. K. (1975) Hydroxyproline excretion in patients with breast cancer and response to treatment. *Br. Med. J.*, ii, 164.

Reynoso, G. (1973) Biochemical tests in cancer diagnosis. In *Cancer Medicine*. (Eds. Holland & Frei). Philadelphia: Lea & Febiger, p. 345.

Snedecor, G. W. & Cochran, W. G. (1967) In *Statistical Methods*, 6th edn, Iowa: State University Press. p. 160.

Urquhart, N. S., Wilks, D. S. & Henderson, C. R. (1973) Estimation associated with linear models: A revisitation. *Commun. Stat.*, **1**, 303.